# Transfer of efficient anti-melanocyte T cells from vitiligo donors to melanoma patients as a novel immunotherapeutical strategy

**DOI:** 10.1186/1740-2557-2-7

**Published:** 2005-08-31

**Authors:** Belinda Palermo, Silvia Garbelli, Stefania Mantovani, Claudia Giachino

**Affiliations:** 1 Experimental Immunology Laboratory, IRCCS Maugeri Foundation, Pavia, Italy; 2 Department of Clinical and Biological Sciences, University of Turin, Turin, Italy

## Abstract

**Background:**

Vitiligo is a relatively common progressive depigmentary condition that is believed to be due to the autoimmune-mediated loss of epidermal melanocytes. High frequencies of self-reactive T lymphocytes directed toward melanocyte differentiation antigens are found in vitiligo patients and might be directly responsible for the pathogenesis of the disease. An interesting aspect of vitiligo is its relation to melanoma: cytotoxic T lymphocytes directed to self antigens shared by normal melanocytes and melanoma cells are found in both conditions, but the resulting immune reactions are completely different. From this standpoint, the selective destruction of pigment cells that occurs in cases of vitiligo is the therapeutic goal sought in melanoma research.

**Presentation of the hypothesis:**

Our working hypothesis is that vitiligo patients might represent a unique source of therapeutic cells to be used in allo-transfer for HLA-matched melanoma patients. The adoptive transfer of *ex-vivo *generated autologous tumor-specific T cells is a therapy that has met with only limited success, essentially because of inability to isolate therapeutically valuable T cells from the majority of tumor patients. Ideally, model systems where strong and efficient responses against the same (tumor) antigens are achieved would represent a better source of therapeutic cells. We believe it is possible to identify one such model in the melanoma-vitiligo dichotomy: T lymphocytes specific for different melanocyte differentiation antigens are found in vitiligo and represent the effective anti-melanocyte reactivity that is often ineffective in melanoma.

**Testing the hypothesis:**

Melanocyte-specific T cell clones can be isolated from the peripheral blood of vitiligo patients and tested for their capacity to efficiently expand *in vitro *without loosing their cytotoxic activity and to migrate to the skin. Cytotoxicity against melanoma patients' non-tumor cells can also be tested. In addition, it would be interesting to attempt an in vivo animal model. If the results obtained from these validation steps will be satisfactory, it might be possible to plan the clinical grade preparation of relevant clones for transfer.

**Implications of the hypothesis:**

When translated into a clinical trial, the possibility of *in vitro *selecting few effective tumor-specific T cell clones for infusion, inherent with this approach, could enhance the therapeutic graft-versus-tumor effect while possibly decreasing the risk of graft-versus-host disease.

## Background

Vitiligo is a common skin disease characterized by the development of white macules and patches associated with local melanocyte loss [1]. Its etiology is not completely known, but the observation of circulating antimelanocytic antibodies and of lymphocytic infiltrations at the margins of lesions in the majority of patients has lent support to the hypothesis that it is an autoimmune disease [2-5].

Many self proteins expressed by melanocytes in the skin of healthy donors, in non-depigmented skin of vitiligo patients and in melanoma patients are demonstrably immunogenic [6-8]. Antigens present in both tumor cells (melanoma) and their normal cellular counterpart (melanocytes) are known as melanocyte differentiation antigens. Among these, Melan-A/MART-1 is a melanosomal protein whose immunodominant epitope for HLA-A*0201 was identified by a screening with cytotoxic T cells [9].

High frequencies of melanocyte-specific CD8^+ ^T lymphocytes are found in vitiligo patients. Using HLA/epitope tetramers, an instrument for measuring the frequency of antigen-specific T cells independently of their functional state [10], Ogg and co-authors [11] directly demonstrated for the first time the presence of high frequencies of CD8^+ ^T cells specific for melanocytic antigens in the peripheral blood of HLA-A*0201^+ ^patients with autoimmune vitiligo. Following this pioneering study, other groups [reviewed in [12-14]] contributed, through the use of tetramers directly *ex vivo*, without any antigen-specific stimulation, to the demonstration that melanocyte-specific CD8^+ ^cells are present in the peripheral blood of both melanoma and vitiligo patients. In particular, high numbers of Melan-A/MART1-specific cells were detected in the majority of patients. Besides circulating cells, melanocyte-specific CD8^+ ^T lymphocytes were also observed *in situ *in both depigmenting lesions of patients with vitiligo [15,16].

These melanocyte-specific CD8^+ ^T Lymphocytes might be relevant for the pathogenesis of vitiligo. The first suggestions came from rare case reports on inflammatory vitiligo [17,18] and immunohistochemical studies later on confirmed the presence of infiltrating T cells in apposition to perilesional melanocytes [19]. Importantly, similar *in situ *T cell infiltrates were also detected in the more common form of the disease, generalized vitiligo [20-22]. Further indications favoring a pathogenetic role for melanocyte-specific, CD8^+ ^T cells in vitiligo came from the direct correlation between their frequency within the total T cell pool and disease activity [11,23], as well as from their capacity to kill HLA-matched tumor cells [11,24,25] and, most notably, normal matched melanocytes [16,26].

Melanoma is an aggressive form of tumor whose incidence increases by 5% per year. Although the presence of melanoma-specific CTLs in cancer patients demonstrate that tumor cells may not completely evade immune recognition, the patient's immune system can only rarely counteract tumor growth [27-29]. An unusual facet of vitiligo is its relation to melanoma: cytotoxic T lymphocytes directed at self antigens shared by normal melanocytes and melanoma cells are found in both conditions and suggest a breakdown of tolerance [11,30-34], yet the resulting immune reaction is the opposite [35-37]. In vitiligo, natural immune tolerance is over-ridden such that the host immune system can orchestrate melanocyte destruction, whereas in melanoma, an immune effector function of potential benefit to the host, i.e., efficient destruction of transformed melanocytes, does occur very rarely. In this respect, it would seem that reactivity to vitiligo melanocytes may be the effective variant of an immune response often ineffective in melanoma. The mechanisms causing these opposite effects are not known but these data, together with the resistance of melanoma to conventional chemotherapeutic and radiotherapeutic approaches, have made the melanoma/vitiligo dichotomy an important model for immunologic investigation.

Avidity of antigen recognition is an important feature of tumor-specific T lymphocytes, determining their capability to kill tumor cells. Notably, a few recent studies indicated that Melan-A/MART1-specific CD8^+ ^T cells isolated from vitiligo patients possess an increased avidity and exert a superior anti-tumor activity than those from melanoma [24,25]. Further indications come from animal models. In one recent study [38] MT-ret transgenic mice, a model for human cutaneous melanoma, were used to investigate the natural anti-tumor T cell response. A large proportion of these animals developed melanoma-associated vitiligo and a good correlation was found between vitiligo development and melanoma control. Interestingly, T cells that secreted IFN-γ in response to melanoma cells were statistically more frequent in melanoma mice that developed vitiligo than in mice that did not, suggesting that vitiligo-associated T cells possessed an increased functional avidity. These data suggest that a qualitative difference exists between the anti-melanocyte cytotoxic T cell responses found in vitiligo versus melanoma that might explain the opposite immunologic outcomes.

## Presentation of the Hypothesis

Autoimmune conditions stem from a break of tolerance to defined autoantigens and this allows for the production of high avidity antigen-specific responses. If these antigens are also relevant to tumor immunity, autoimmune cells can be exploited for tumor intervention. Our working hypothesis is that vitiligo patients might represent a unique source of therapeutic cells for HLA-matched melanoma patients, essentially due to their superior TCR affinity and increased tumoricidal potential.

The adoptive transfer of *ex-vivo *generated autologous tumor-specific T cells is a potentially potent therapy that has met with only limited success. An important limitation to such treatment is inability to isolate and generate therapeutically valuable T cells from the majority of tumor patients. The causes of these limitations are not well known, but might include the inability of the tumor cells to trigger a T-cell response (ignorance) or their ability to actively suppress or delete T cells of the highest avidity (tolerance). In addition, T cells derived from melanoma patients may often display functional impairments as a consequence of the tumor environment, which inhibits their acquisition of final effector functions [32]. Indeed, i*n vitro *culture of these tumor-sensitized T cells with appropriate activation stimuli has been shown to partially restore normal functional properties [39] and to confer effector activity that could potentially sustain tumor rejection upon re-infusion [40]. However, in the majority of cases, autologous T cells are found to exert only poor cytotoxic activity toward HLA matched melanoma cells. On the other hand, CTLs against different melanoma associated antigen are found in vitiligo and mediate autologous melanocyte destruction [24-26,30,31]. Notably, the Melan-A/MART1-specific CD8^+ ^T cells isolated from vitiligo patients appear to possess an increased avidity and to exert a superior anti-tumor activity than those from melanoma [24-26].

How can this knowledge be applied to the definition of new immunotherapeutic strategies for melanoma patients? A first possibility is an allo-transfer approach, where very efficient anti-tumor CD8^+ ^T lymphocytes from vitiligo donors can be transferred into HLA-matched melanoma patients. High-avidity and tumor-specific T cell clones can be isolated from the peripheral blood of HLA-A2 vitiligo patients; if these could be proven to efficiently expand *in vitro *without loosing their cytotoxic activity and to maintain their skin-homing capability, they might represent a valuable source of therapeutic cells.

## Testing the Hypothesis

In order to test the hypothesis, melanocyte-specific T cell clones could be tetramer-sorted from the peripheral blood of vitiligo patients and tested for their capacity to efficiently expand *in vitro *without loosing their cytotoxic activity. They will also be tested for their skin-homing activity by FACS-evaluating surface expression of the cutaneous lymphocyte antigen, CLA. As a first effort in determining the effects of host cellular environment on allo-transfer, these cells can be mixed with PBMC from HLA-matched melanoma patients and the anti-tumor potential of the vitiligo/melanoma pools assayed. Possible immunosuppressor effects exerted by either the melanoma patients' sera [41] or the tumor itself might be addressed through co-cultures. Cytotoxicity against melanoma patients' non-tumor cells (peripheral blood, skin biopsies) can also be tested. In addition, it would be interesting to attempt an in vivo animal model using immunodeficient SCID or RAG knockout mice, rendered transgenic for human HLA and transplanted with melanoma. These mice might be treated with the clone from a vitiligo patient, with or without melanoma patient's lymphocytes. If the results obtained from both the in vitro validation step and the in vivo animal model will be satisfactory, it will be possible to plan the clinical grade preparation of relevant clones for transfer. A major drawback of this approach remains achievement of HLA match between a T cell donor and a melanoma patient. Only weak associations between vitiligo and several HLA-class I and -class II alleles have been reported to date, and essentially no significant association exists with melanoma. So, as patients with vitiligo represent about 1% of the general population, the probability to find a perfect match can be estimated to be a hundred-time lower than the one theoretically achieved for allogeneic transplantation involving the entire population as potential donors.

## Implications of the Hypothesis

If this hypothesis were true, at least some autoimmune diseases might be seen as novel sources of therapeutic cells for tumor patients. When translated into a clinical trial, the possibility of *in vitro *selecting few tumor-specific T cell clones for infusion, inherent with this approach, could enhance the therapeutic graft-versus-tumor effect while possibly decreasing the risk of graft-versus-host disease.

## Competing interests

The author(s) declare that they have no competing interests.

## Authors' contributions

B.P. participated in the design of the study; S.G. and S.M. participated in analysis and interpretation of data; C.G. conceived the study and drafted the manuscript.
